# Discrepancies in infant feeding recommendations between grandmothers and healthcare providers in rural Mexico

**DOI:** 10.1186/s13006-022-00518-0

**Published:** 2022-11-22

**Authors:** Paulina Luna, Nerli Paredes-Ruvalcaba, Tania Valdes, Barbara Guerrero, Angélica García-Martínez, Rafael Pérez Escamilla, Diana Bueno-Gutiérrez

**Affiliations:** 1 Yale University School of Medicine, New Haven, CT USA; 2 Department of Anthropology, Michigan State University, East Lansing, MI USA; 3 Facultad de Psicología, Universidad del Desarrollo, Santiago, Chile; 4 Servicios Integrales de Atención en La Infancia, Ciudad de Mexico, México; 5 Lucy Family Institute for Data and Society, University of Notre Dame, Notre Dame, IN USA; 6 School of Public Health, Yale University, New Haven, CT USA; 7 Facultad de Medicina Y Psicología, Universidad Autónoma de Baja California (UABC), Tijuana, BC México

**Keywords:** Breastfeeding, Qualitative, Socio-ecological framework, Indigenous communities, Coloniality of biomedicine

## Abstract

**Background:**

Infant feeding practices are rapidly changing within rural areas in Mexico, including indigenous communities. The aim of this study was to compare infant feeding recommendations between grandmothers and healthcare providers, to better understand the factors that may influence these practices within these communities. This study builds on research that recognizes the legacy of colonization as an ongoing process that impacts the lives of people through many pathways, including the substandard healthcare systems available to them.

**Methods:**

Qualitative study based on secondary data analysis from interviews and focus groups guided by a socioecological framework conducted in 2018 in two rural, Indigenous communities in Central Mexico. Participants were purposively selected mothers (*n* = 25), grandmothers (*n* = 11), and healthcare providers (*n* = 24) who offered care to children up to two years of age and/or their mothers. Data were coded and thematically analyzed to contrast the different perspectives of infant feeding recommendations and practices between mother, grandmothers, and healthcare providers.

**Results:**

Grandmothers and healthcare providers differed in their beliefs regarding appropriate timing to introduce non-milk foods and duration of breastfeeding. Compared to grandmothers, healthcare providers tended to believe that their recommendations were superior to those from people in the communities and expressed stereotypes reflected in negative attitudes towards mothers who did not follow their recommendations. Grandmothers often passed down advice from previous generations and their own experiences with infant feeding but were also open to learning from healthcare providers through government programs and sharing their knowledge with their daughters and other women. Given the contradictory recommendations from grandmothers and healthcare providers, mothers often were unsure which advice to follow.

**Conclusions:**

There are important differences between grandmothers and healthcare providers regarding infant feeding recommendations. Healthcare providers may perceive their recommendations as superior given the neocolonial structures of the medical system. Public health policies are needed to address the different recommendations mothers receive from different sources, by harmonizing them and following an evidence-informed approach. Breastfeeding programs need to value and to seek the participation of grandmothers.

**Supplementary Information:**

The online version contains supplementary material available at 10.1186/s13006-022-00518-0.

## Background

The benefits of breastfeeding are well established and are particularly heightened in environments that have been economically and socially marginalized, where it is crucial to protect infants from infectious diseases and malnutrition [1–3]. Exclusive breastfeeding is key in reducing under-five mortality, improving human development, and supporting environmental sustainability. Therefore, increasing exclusive breastfeeding rates is essential for meeting the 2030 Sustainable Development Goals (SDG) [4], particularly those related to food security, maternal and child health, economic growth, and sustainable consumption.

In Mexico, the reported rates of exclusive breastfeeding (EBF) during the first six months and the mean duration of breastfeeding have been increasing, according to the Health and Nutrition National Survey (ENSANUT). Exclusive breastfeeding is defined as feeding infants only with breastmilk and no other fluids or food except medicines, based on recalling the consumption of a list of food and beverage items during the previous 24 h. In 2014 the mean duration of breastfeeding was 8.8 months [5] and increased to 9.8 months in 2018 [6]. Regarding exclusive breastfeeding (EBF) during the first six-months of age, rates went from 14.4% in 2012 to 28.6% in 2018 [7]. Despite this increase, the use of infant formula remains high, with 42.9% of infants being fed infant formula by 12 months of age. While this percentage is higher in urban areas (47.6% vs. 31.4% in rural areas) and in non-Indigenous communities (44.8% vs. 22.9% in Indigenous communities), it is well known that the formula industry continues to expand rapidly across highly vulnerable rural and Indigenous communities [8–10].

In Mexico, consistent with a socioecological model, multiple factors that impact breastfeeding rates and duration have been identified at the individual, group, and structural level [11]. Factors at the individual level include pain, perception of insufficient milk, lack of time, and discomfort. Group level factors include lack of family, employers and healthcare providers (HCPs) support. At the structural level the following factors have been identified: marketing of infant formula, social norms associated with “modernity” that promote the use of infant formula, and the social pressure against breastfeeding in public [7, 8, 12–15].

As previously documented [12–15], social and cultural changes in rural, Indigenous communities in Mexico are associated with ideas of modernity and development, leading to increased infant formula use and decline in exclusive breastfeeding. This work has also shown how infant formula companies penetrate the healthcare system via HCPs engaged with maternity services.

Additionally, research has revealed that grandmothers are role model figures who have a strong influence on mothers’ infant feeding practices [16–18]. Studies have found positive effects on EBF by including grandmothers in breastfeeding promotion programs [19, 20]. This impact happens in spite of the fact that their knowledge and perspectives are often dismissed, rejected, or perceived as backwards by public health initiatives that seek to increase breastfeeding rates [18, 21].

The majority of public health efforts designed to increase breastfeeding rates in Mexico tend to focus on biomedical approaches and rarely take into account the role of sociocultural influences. This is problematic, as previous research has reported mismatches between mothers and HCPs’ perspectives of breastfeeding stemming from sociocultural factors [22]. For example, public health efforts that primarily focus on telling mothers the health benefits of breastfeeding may not be giving enough importance to the key need to address the sociocultural issues that mothers perceive as influencing their breastfeeding practices. Therefore, a mother may know the health benefits of breastfeeding and intend to breastfeed, but lack of maternity leave or other work accommodations may prevent her from breastfeeding. Furthermore, ignoring the community perspective has resulted in unintended negative consequences that can end up damaging breastfeeding rates or are simply irrelevant to the sociocultural context in which they operate. For example, research with a Nahuatl Indigenous population in Morelos, Mexico [13] revealed that programs promoting breastfeeding in this population were unnecessary since the majority of mothers felt confident in their knowledge and had successful strategies to meet their breastfeeding goals. Additionally, it was reported that comments from HCPs actually discouraged mothers from breastfeeding.

A commonly used public health strategy to increase breastfeeding rates is through HCPs’ training. Unfortunately, most of these interventions are reduced to theoretical information without clinical time to apply this knowledge and are oftentimes sponsored by infant formula companies [23]. These programs do not offer the needed space and time to address authoritative, classist, racist, and sexist practices within the medical system that have resulted from the colonial vision of biomedicine [24]. For example, during individual interactions, HCP’s tend to impose their knowledge (or lack thereof) about breastfeeding practices without considering the voice and experiences of mothers and grandmothers [13, 22]. As a result, health inequities are further reproduced, especially among Indigenous communities.

Therefore, the objective of this study was to compare infant feeding recommendations between grandmothers and HCPs within the context of rural, Indigenous populations in central Mexico. Analyzing these two perspectives provides the opportunity to obtain a more nuanced understanding of the multiple factors that impact differences in infant feeding perspectives and the dilemma it may represent for mothers. Additionally, this study examined how mothers responded to the opinions and recommendations received from both grandmothers and healthcare professionals. For our analysis, we build on previous research that highlights Indigenous and non-Western perspectives [25–27] in the context of colonization [28]. We expect that our findings will be of significance as the effects of colonization are ongoing and continue to impact various aspects of Indigenous peoples’ lives, including the healthcare they receive [29]. Moreover, Indigenous mothers and grandmothers have been historically the primary targets of the modernizing agenda and their knowledge is often perceived as backwards [30].

## Methods

This study was a secondary analysis of data available from a cross-sectional qualitative study conducted in 2018 by our team that was informed by a socioecological framework (SEF) [12].

### Study sites

This study took place in the State of Mexico, one of country’s 32 states, which is situated at the center of the nation and has a population of almost 15 million people, of which 9.1% identify as Indigenous [31]. The State of Mexico faces high rates of inequity as indicated by the Gini coefficient of 0.42 [32]. This study was conducted in the communities of Ganzdá and Santa Ana Nichi, which were chosen as study sites through a partnership with Un *Kilo de Ayuda* (UKA), a national non-for-profit civil society organization focused on early childhood development that had previously worked with both communities. Ganzdá has a population of approximately 2400 inhabitants, of which 34% identify as Indigenous, the majority as Otomí; Santa Ana Nichi has a population of about 1,200 people, of which 26% identify as Indigenous, mostly Mazahua [26, 27]. At the time of the study (2018), approximately 75% of people in Ganzdá and Santa Ana Nichi were affiliated with *Seguro Popular*, a social government program that provided healthcare services for people without formal employment, i.e., they had informal employment or were unemployed [26]. In addition to *Seguro Popular*, women in these communities had access to perinatal healthcare services through *Prospera’s* conditional cash transfer program (CCTP), private clinics (some affiliated with pharmacies), and traditional healers. *Seguro Popular* and *Prospera* were terminated after the present study was conducted. The *Instituto de Salud para el Bienestar (INSABI, Institute for Health and Well Being)* was created to replace the *Seguro Popular* as a different program to which all people from Mexico can register to receive health care, but mainly continues to serve people who may not have formal employment or health insurance through their employer.

### Design and participant selection

Participants in the original study included mothers, fathers, grandmothers and grandfathers of children two years of age or younger, and HCPs who provided services to mothers and children of this same age group (physicians, nurses, health workers and traditional healers). Recruitment for family members was conducted through purposive sampling by approaching potential participants in areas where people congregate (i.e., open markets, public squares, waiting areas in clinics). HCPs were recruited at all health centers and clinics in each community by speaking with the director at each site and coordinating meetings with providers who met the above inclusion criteria. Given that the present study focused on the perspectives on infant feeding of mothers, grandmothers, and HCPs, only data from these three groups was analyzed.

### Focus groups and interviews

For the original study, four female researchers (PL, BG, TV and DB) conducted semi-structured interviews and focus groups in Spanish between June and August 2018. Interviewers were native Spanish speakers trained in qualitative research methods. The durations of interviews ranged between 20 min to one hour, while focus groups lasted between 40 and 50 min. A demographic questionnaire was administered at the end of the interviews and focus groups. The number of interviews and focus groups conducted was enough to reach data saturation. It was determined that saturation had been reached once major, codebook-based themes started to become repetitive.

### Script guide

The guide for interviews and focus groups was based on a SEF and further reviewed for cultural appropriateness by our collaborators at UKA. Guides differed between mothers, grandmothers, and HCPs, with variations applicable to each group. However, all guides covered topics related to: (1) prenatal experiences; (2) infant feeding practices before/after 6 months postpartum; (3) infant feeding practices in the community; (4) benefits of breastfeeding and (5) work and school. The guide for grandmothers focused on their daughter or daughter-in-law’s experiences with pregnancy and infant feeding. The guide for HCPs additionally explored: (1) protocols surrounding pregnancy and deliveries; (2) breastfeeding recommendations and (3) formula industry marketing practices. The interview guides can be found in the Additional file 1.

### Data analysis

Interviews and focus groups were recorded and transcribed verbatim by native Spanish speakers. Researchers took notes of the interviews for participants who did not wish to be recorded. All the recordings, notes, and transcripts were reviewed by PL for accuracy and quality.

Two authors (DB and NPR) used thematic analysis [33, 34] to explore the different perspectives of infant feeding recommendations and practices between mothers, grandmothers, and HCPs. All interviews were read multiple times by both coders to develop a consensus codebook using an inductive process. Key quotes were selected per coded theme. Both authors wrote notes throughout the coding process and resolved discrepancies through consensus building discussions. Codes were then abstracted and organized into main themes and sub-themes. PL reviewed all the themes and subthemes, and consensus between these three authors was achieved through discussion. All data analysis was done in Spanish to maintain meaning.

Strategies for data triangulation included [35]: (1) analyzing information obtained from the perspectives of mothers, grandmothers, and HCPs on infant feeding practices and recommendations; (2) utilizing different methods for data collection through interviews and focus groups and (3) having two data coders and one reviewer/moderator with different backgrounds (medicine, community nutrition, and anthropology).

## Results

In total, we used 51 transcripts of conversations with 60 participants. We used 21 transcripts for conversations with 25 mothers (one focus group with five participants, two combined interviews with a mother and grandmother, and 18 individual interviews); eleven transcripts of conversations with eleven grandmothers (two combined interviews with a mother and grandmother and nine individual interviews); and 19 transcripts of conversations with 24 HCPs (one focus group with six participants, one interview with two participants, and 16 individual interviews). A summary of participants’ sociodemographic characteristics can be found on Tables 1 and 2.

**Table 1:** Sociodemographic characteristics of mothers and grandmothers from Indigenous communities, Santa Ana Nichi and Ganzdá, Mexico

	**Mothers**		**Grandmothers**
	Santa Ana	Ganzdá	Santa Ana
	(*n* = 14)	(*n* = 11)	(*n* = 11)
**Age (years), median (range)**	23 (17–29)	27 (16–31)	48 (38–54)
**Education, n (%)**^a^			
Elementary school	4 (29)	1 (17)	7 (64)
Middle school	6 (43)	5 (83)	3 (27)
High school	2 (14)	-	-
College	2 (14)	-	-
Did not attend	-	-	1 (9)
No information	-	5	-
**Occupation, n (%)**^a^			
Homemaker	8 (62)	5 (100)	8 (73)
Business	4 (31)	-	3 (27)
Student	1 (7)	-	-
Skilled worker	-	-	-
No information	1	6	-
**Marital status, n (%)**^a^			
Married	3 (21)	2 (40)	6 (55)
Living with partner	11 (79)	3 (60)	3 (27)
Single	-	-	2 (18)
No information	-	6	-
**Speak/Understands Indigenous language, n (%)**^a^			
Yes	6 (43)	1 (20)	4 (36)
No	8 (57)	4 (80)	7 (64)
No information	-	6	-
**Recipient of social program, n (%)**^a^			
Yes	5 (36)	3 (60)	9 (82)
No	9 (64)	2 (40)	2 (18)
No information	-	6	-

^a^Percentage is for individuals with known information

**Table 2:** Sociodemographic characteristics of healthcare providers from Indigenous communities, Santa Ana Nichi and Ganzdá, Mexico

	**Santa Ana**	**Ganzdá**
	(*n* = 14)	(*n* = 10)
**Age (years), median (range)**	31 (25–52)	23 (22–46)
**Sex, n (%)**		
Female	7 (50)	9 (90)
Male	7 (50)	1 (10)
**Title, n (%)**		
Medical doctor	8 (57)	1 (10)
Pediatrics	1 (12^a^)	0^a^
Obstetrics/Gynecology	3 (38^a^)	0^a^
General medicine	4 (50^a^)	1 (100^a^)
Nurse	4 (29)	9 (90)
*Hierbera* (Healer)	1 (7)	-
Health promoter	1 (7)	-
**Time practicing (years), median (range)**		
	0.5 (8 days-20 years)	12 (0.5–19)
**Time in current community (years), median (range)**		
	3.25 (0.5–31)	4 (5 months-19 years)

^a^Percentage amongst medical doctors

We identified five major themes through the qualitative data analysis. The first two themes, “Infant feeding perspectives from grandmothers” and “Infant feeding recommendations from HCPs,” provide a general overview of the perspectives and recommendations that these two groups had on infant feeding practices. The third theme, “Who do mothers listen to?” explored how mothers responded to receiving conflicting recommendations. The fourth theme, “Tensions between perspectives from grandmothers and HCPs: the role of traditional and modern infant-feeding models,” compared the major differences between the perspectives of both groups. The last theme, “Maternal blame and suffering,” emerged by identifying some of the similarities between infant-feeding perspectives from both grandmothers and healthcare professionals.

### Infant feeding perspective from grandmothers

Data from the interviews revealed that perceptions of grandmothers regarding ideal infant feeding practices sometimes differed from recommendations from HCPs. These differences were more noticeable regarding the early introduction of herbal teas, particularly to help alleviate colic pain. Some grandmothers were aware that such practices were not recommended by HCPs, nonetheless, given their own experience using teas as home remedies, they recommended giving tea to infants to improve their health:

*"Well, I also gave them star anise for their colic pain, but right now the pediatrician said no. That it is very dangerous because they could even die, he says. But I gave all my children star anise.”* (Grandmother 2)

*“No, just breast milk. Because supposedly you cannot give it [tea] to the baby, although I once did tell her to give her a tea because the girl was very constipated with her own milk. The pediatrician told us that it was normal because it was in colostrum, but I told her that nothing would happen to her if she gave her water since I raised them that way. And the girl is there, perfectly fine.”* (Grandmother 3)

Some grandmothers were also aware that introducing solid foods prior to six months of age was not recommended by HCPs. Yet, they stated that allowing infants to taste small quantities of food is an important way for infants to learn to eat a varied diet:

*“The doctors say that they cannot eat because the body or intestines are still not ready for heavy things, but we also did not fill them up, we only gave them little tastes of food. And that's how my children taught themselves to eat everything. In fact, I have this child, the youngest, who doesn't eat vegetables for the same reason.* (Grandmother 3)

Another key difference between these groups centered on breastfeeding duration. This was especially important, as culturally, it is common for women to accompany their daughters or daughters-in-law to health visits and hear the HCPs’ recommendations. According to some grandmothers, HCPs recommended discontinuing breastfeeding at six months postpartum because they believed human milk is no longer nutritious or useful to the infant after that time. However, grandmothers encouraged breastfeeding up to two years of age or beyond. For example, one grandmother shared her experience of accompanying her daughter to the clinic.

*"Q: And more or less, at what age is [the BF] stopped?*

*A: Well, they have told us that at 6 months we should stop breastfeeding, but we can breastfeed for longer, for like 2 years.*

*Q: And did they also tell you that at the clinic, or some doctor?*

*A: No, here [at the community clinic] they told us that only up to 6 months, but basically it is up to us to do it, up to 2 years [laugh]. Or two and a half years.”* (Grandmother 4)

Beyond being aware of the infant feeding recommendations differences between themselves and HCPs, grandmothers also expressed being aware of how their recommendations may be perceived by mothers. Mainly, some grandmothers noticed that mothers were more likely to first follow recommendations from HCPs. There were also occasions in which grandmothers themselves encouraged mothers to obtain their doctor’s advice prior to using traditional or home remedies:

*"Q: Well, what about if you got sick, had “susto” [fright] or “empacho” [digestive problem], what would you recommend to your daughter-in-law?*

*A: I would tell her to first take him to the doctor . . . Follow what the doctor says and if that doesn’t work, then we go to the home remedies we know.”* (Grandmother 2).

Overall, grandmothers were open to learning from HCPs and noticed some changes within the biomedical system to promote and facilitate breastfeeding. Furthermore, they seemed open to learning from HCPs through the government programs available. They were also likely to use the knowledge gained to inform their own perceptions of infant feeding practices and share this knowledge with their daughters and other women:

*“And right now what they are doing in the hospitals is that the baby is born and then later they give it to the mother so that she can breastfeed, so that forces them to breastfeed. And also in the clinics, they no longer let you take a bottle.”* (Grandmother 2)

*Right now, like I’m telling you, I have supported my daughter-in-law and my daughter, because when I brought them to their medical appointments, they [HCPs] explained to them, and I started learning . . . To not give them water before 6 months. Because I used to, when my children got sick or something, I did give them tea or something like that because I did not have the information. Or also, it helped me a lot that when they went to Prospera, there, they gave us talks.* (Grandmother 1)

### Infant-feeding perspective from HCPs

HCPs tended to make a clear division between their infant feeding recommendations and the ones given by grandmothers and anyone outside the biomedical field. HCPs perceived their recommendations as superior to the recommendations by grandmothers. Some HCPs also expressed negative attitudes towards mothers and the importance of removing ideas that were not in line with their biomedical thinking:

*“Oh yeah. Mothers-in-law and grandmothers. It’s because the area where we live is kind of like that, low class. So women are clinging to what their grandmother told them, to what the other grandmother told them, and to what the one who is already dead said, and to what the great-great-grandmother said.”* (Nurse 1)

*"It’s rare for me, I mean, eh, they don't talk so much about it (Practices like evil eye), and maybe because from the beginning we tried to make them understand that it doesn't exist, you know?”* (Pediatrician 1)

*"Before doing any intervention, you have to know what they think to remove those ideas they have . . . And remove any of their ideas or stereotypes that they have.”* (Physician/General practitioner 1)

Some HCPs also mentioned the idea that mothers were somehow easily malleable entities that followed any advice given to them:

*“They let themselves be guided more by what people tell them, by what they see, or by something easier, than by what it is . . . Basically, well, their beliefs tell them . . . Not what a doctor tells them, not what a pediatrician tells them, it's almost like they question what we tell them, you know? It’s like [they follow] only to their own culture, you know? It’s how they are educated, nothing else, that’s only what they respond to.”* (Physician 1)

*“Educating new moms is very difficult because they are already well practiced from their house, from their aunt, from their I-don't-know-who. ‘They told me that I should drink a lot of atole,’ for example in this case, they drank a lot of atole because by taking atole, they produced milk and gave their baby. Well bad. The atole is nothing but pure tortilla, and it is the same”* (Nurse 1).

### Who do mothers listen to?

Mothers expressed that they consider the opinions of both grandmothers and HCPs. The advice they ultimately follow largely seems to depend on the context and what fits their immediate needs. In the following example, a mother decided to follow her own mother’s advice to breastfeed for two years, even though the doctor had told the mother that her breast milk was no longer useful:

*"Q: And did someone advise you to keep [breastfeeding] until two years of age?*

*A: Well it was really our beliefs, according to them it is necessary to give [breast milk] up to 2 years.*

*Q: And who told you?*

*A: Ah, my mom.*

*Q: And who made you this comment, this suggestion that it is no longer good after 6 months?*

*A: Ah, the doctor up here.”*

(Mother 1)

The following example illustrates a mother who decided to follow the advice from a HCP. This mother expressed that she had given herbal teas to her older children prior to them being six months of age. Nonetheless, she decided to not do that with her youngest infant given that both her husband and doctor advised her not to do it:

*"Q: Ok. And did you give them the teas that your mother used to tell you?*

*A: No, uh, for my other girl I did, but then, mmh, my husband didn't want me to. Eh, because it could maybe be harmful and so.*

*Q: Mjm, ok. So you more... uh, well, you prefer to stick to what the pediatrician tells you, what the doctors tell you, in the program... ok.*

*A: Yes.”*

(Mother 2)

## Tensions between perspectives from grandmothers and HCPs: the role of traditional and modern infant feeding models

Comparing the perspectives of grandmothers and HCPs indicated that these groups subscribe to distinct infant-feeding models. Grandmothers’ perspectives were more in line with traditional models that were perceived as “old/outdated” by HCPs. Meanwhile, HCPs perspectives were aligned with “modern” models associated with rationality, science, and autonomy. This conflict was made more evident from the perspective of HCPs, who expressed frustration when mothers did not follow their recommendations. From their perspective, mothers who did not follow their advice were associated with ignorance and ineptitude. These negative stereotypes reflected HCPs’ racist, classists, and sexist ideas.

*“People don’t understand due to their ignorance. We insist to them, but they still give tastes of meat, coke, teas [to their infants]. If only I could give you the list of what [food] they give. But people prefer to watch their soap operas or soccer.”* (Gynecology Resident 1)

*“The ideology that they have. Like they do not want to get out of what is ignorance, it’s like, one explains to them and nothing. They are guided more by what people tell them than by what one is explaining to them.”* (Physician/ Medical practitioner 1)

In other instances, HCPs perceived mothers as being influenced too much by media’s promotion of infant formula and processed infant food. From their perspective, this was an important contributor of why mothers did not follow their recommendations. Once again, not following their advice was associated with negative stereotypes:

*“Well here, in the community, a lot! [Influence of the advertisement of baby food products] . . . Because, for example, the ones that are advertised on television, those are the ones that moms look for the most. And in fact, not only in terms of formulas, even in terms of baby food.”* (Physician/ Medical practitioner 2)

*“Many times because of the mother's laziness, the most feasible thing seems to be the bottle, if the baby starts crying at home and the mother is doing her activities, it makes things easier for her to just give formula, Gerber [brand of baby pureed food] and even Danonino [fermented yogurt marketed for young children] . . . All with the intention to just quiet the baby. They do it unconsciously without realizing the damage they are causing them.”* (Health Promoter)

From the perspective of grandmothers, there are important generational changes taking place that seem to be at least in part due to mothers wanting to align themselves with Western beauty standards, and not wanting their bodies to change because of breastfeeding. This, according to some grandmothers, led mothers to not want to breastfeed, which was also conflated with being lazy or not maternal enough:

*"Because right now the moms of today are more modern, they no longer want their breasts to be disfigured, or they don't like to lose their figure, because for example, a modern mom now doesn’t want their belly to come out, or sometimes either, it is already happening that they do not want to get pregnant, because [they say] I do not want to lose my figure and that my husband will no longer accept me . . . I tell my daughter-in-law, ‘You are now all lazy.’”* (Grandmother 1)

*“Many of the girls no longer study and go to work in the city. And then obviously from the city they adopt, other things, other behaviors. And since they see that their employers use formulas there, they prefer to not give breast milk anymore. But then they get married, and the mother-in-law will always be, or the mother, demanding that they have to give them breast milk because if not, then they are considered to be not real mothers, ‘mothers’, just like, well you did raise them, but it’s not how a woman from here should be.”* (Hierbera/Traditional Healer)

### Maternal blame and suffering

We found that many grandmothers and HCPs perceived mothers as the sole individuals who should carry the burden of ensuring that infants were fed appropriately. This pressure placed on mothers disregards the physical and emotional burden that many mothers experience when trying to implement their infant feeding choices, including breastfeeding, without the support of other family members.

From the perspective of grandmothers, there was the added pressure that motherhood came with suffering and refusing to suffer was perceived as a negative character trait:

*“Well, I say that it is because they say, oh it hurts me, it’s because it hurts my breasts, it’s like, how do you think I am going to give it, you give them, it’s what I have heard, vulgarly, you know? I grabbed that freaking kid that already hurt my breast. And like I told you, right now with my daughter-in-law with this baby that she had, there are already 3, her breasts are cut and with so many cracks, I told her that this is normal, and you have to give them [breast milk] because that is how it is . . . And that is why they prefer to give them milk with the bottle . . . And so, I tell her, you have to suffer. And it’s not my fault [laughs]”.* (Grandmother 5)

From the perspective of HCPs, there was the perception that mothers are not fully committed to their infants if they do not feed according to their recommendations:

*“If you really do not want to give your baby [breast milk], it is because you do not love them. That’s the truth. Because how hard can it be for you? On those days you are going to be resting, you are not going to work, you are going to be with your baby all day and that is the only thing you are going to do.”* (Nurse 1)

*“It enters them on one side and leaves from the other” because they do not have the commitment to be able to tell you yes or no . . . Many times due to the mother's laziness, the most feasible option seems to be the bottle.”* (Health Promoter)

## Discussion

The results from this study indicated that there are important differences between grandmothers’ and HCPs’ infant feeding recommendations. These differences are important to consider because they may instigate tension and conflict as mothers decide how to feed their infants.

### The role and perspectives of grandmothers on infant feeding

Grandmothers can be either great allies or great adversaries to breastfeeding [16–20]. In our study, some grandmothers were open to learning from HCPs through workshops offered by government programs and they were likely to share the knowledge they gained with other women. It is important to note, however, that there were some key differences between the perspectives of grandmothers and HCPs. Specifically, some grandmothers expressed the importance of giving herbal teas to infants. Others favored the early introduction to solid foods, and some expressed the value of breastfeeding well beyond six months postpartum.

Previous studies have reported the circumstances in which mothers may be more likely to follow the advice of grandmothers regarding infant feeding practices. A meta-ethnographic study with migrant women´s experiences of breastfeeding, reported that mothers were more likely to take into consideration the opinion of grandmothers if they lived with them or maintained a close relationship with them [36]. Results from another study in a Triqui community in Mexico further suggested that when mothers were experiencing vulnerable moments, in which they felt insecure, they were more likely to follow recommendations from their mothers to avoid any risks or health complications for their infants [37].

### Biomedicine and colonialism

Healthcare professionals tended to perceive their infant-feeding recommendations as superior to the recommendations of grandmothers and anyone outside the biomedical field. This reproduced a clear hierarchy in which any knowledge outside the biomedical model was disregarded. This hierarchy can be associated with the coloniality of biomedicine, in which the biomedical model superimposes its knowledge as the most valuable and discounts any other knowledge, including that of communities [24–27, 38]. Previous research has shown that HCPs believe they have the absolute truth, and as such, there is no need for them to consider the opinion of women regarding their infant feeding practices [22, 23]. These assumptions lead HCPs to consider themselves as breastfeeding experts and to have the power to decide what is “natural” for mothers and infants [39]. This, in turn, can make doctor-patient communication difficult for mothers, as HCPs try to impose their visions in an authoritarian, vertical, and hierarchical way.

Despite the assumption that the infant feeding knowledge of HCPs is the most accurate, numerous studies have shown that this is not always the case [11, 12, 14, 23, 35, 40]. This can be attributable to several reasons. First, there is a lack of training on how to support breastfeeding mothers [11, 12, 23, 40]. Second, the biomedical model tends to favor the use of breast milk substitutes to improve the "efficiency" of infant feeding and not "waste time" accompanying and helping mothers breastfeed their infants [11, 23]. Third, some HCPs only value the biological and nutritional aspects of breastfeeding and pay little attention to the sociocultural context that impacts infant feeding practices [12–14, 23]. This may make it difficult for HCPs to understand why mothers do not breastfeed even though they may be able to recite the list of nutritional and immunological properties of human milk.

Not understanding or valuing the sociocultural context of infant feeding may be difficult for HCPs as the vast majority that work in rural communities tend to be foreign to the communities they serve [38, 41, 42]. Previous research [42–44] has corroborated that HCPs who perform internships in smaller provinces tended to dismiss traditional concepts and beliefs during doctor-patient interactions. Furthermore, having received training based on a biomedical model makes it difficult for HCPs to understand and identify the complex realities of the communities they serve.

It is unclear why HCPs in our study made the assumption that mothers do not follow their advice, but utilizing a broader perspective that accounts for the ongoing colonial history of Mexico may provide important insights. The biomedical system is a direct result and perpetuator of colonial legacies, including classist, racist, and sexist attitudes [38]. This perspective informs HCPs’ assumption that rural and Indigenous mothers and people in general are too “ignorant” to follow advice from healthcare professionals. Previous research in Mexico has found that HCPs are likely to attribute the lack of adherence to medical recommendations of their Indigenous and poor patients to supposed ignorance and obstinacy [41]. Another study conducted in Mexico also reported that HCPs tended to perceive low-class, dark-skinned (sometimes Indigenous), women as problematic, hyper-fertile, ignorant, filthy, unable to care for children, and that their sole contribution to society was to birth so called “defective” citizens [45].

### The influence of media on infant feeding

Findings from our study indicate that messages from the media may have an impact on infant feeding practices. Interviews from HCPs revealed that some of them perceived that the media was heavily influencing how mothers cared for their infants. For example, some HCPs reported that through media, infant formula was portrayed as a “modern” commodity that makes it easier for mothers to deal with crying infants.

These findings align with previous research conducted in Mexico that reported how human milk substitutes are advertised. Research from a rural Nahuatl community in Morelos reported frequent TV broadcasts of human milk substitutes paired with blond babies from apparently high socioeconomic status [42]. Another study on the marketing of breast milk substitutes further reported that 85% of mothers were exposed to this type of advertising on television. These advertisements were broadcasted mostly in the afternoon and evening, specifically during soap operas and family shows, thought to be the time most preferred by women [46]. An additional study identified breast milk substitute’s promotion in nearly all retail stores, pharmacies, and convenience and corners stores visited [47].

### Maternal blame and suffering

Community support and care for mothers during the postpartum period has been gradually declining within the context of Mexico [48]. Traditionally, women in Mexico received emotional and physical support from the women in their families and communities [48, 49]. Examples of such support women received during the postpartum period included housework, care for other children, and maintaining social networks to promote a sense of belonging to the community. Today, mothers are expected to recover rapidly and return to their “productive” lives. This is further complicated for mothers who work outside the home, as they are expected to fulfill their domestic and parenting “obligations” as well, which imposes a double workload and a significant obstacle to breastfeeding.

In our study we found that grandmothers and HCPs perceive mothers as becoming increasingly irresponsible in their parenting. Mothers who do not breastfeed are seen as lazy, selfish, as not caring for their children, and as only concerned with their body image or social life. Previous research reported that HCPs frequently blamed mothers for neglect or carelessness towards their children when they did not carry out the biomedical recommendations or practices that they assumed they should know and carry out [39]. Another study conducted in Bolivia found that women who received the most criticism were often those with the least power to respond to negative judgments and discrimination, poor and Indigenous mothers [25].

While mothers are blamed and held as the only ones responsible for their infants, ironically, they often experience significant invisibility when communicating with HCPs [22]. It appears that the experiences of mothers are not taken seriously by healthcare professionals. Instead, mothers tend to receive homogeneous and standardized recommendations that ignore their particular contexts [13, 23]. Moreover, mothers often encounter disrespectful attitudes, such as a mother in Bangladesh reporting that a doctor compared her to a cow [39].

The present study found that some grandmothers and HCPs considered pain and suffering as an essential component of motherhood. Other studies in Latin America have also corroborated this finding [22, 25]. A study in Bolivia reported that suffering and sacrifice were perceived as central components of motherhood [25]. Maternal sacrifices start during the prenatal stage when mothers must comply with food cravings for the sake of their babies. The sacrifices continue with the pain of childbirth, which is essential to establish a lifelong bond between mother and child. Suffering is then reinforced through breastfeeding, which is believed to have the possibility of transmitting the emotional states of the mother to the child. Future research should further analyze the effects of these notions around pain with breastfeeding in different contexts in Latin America.

### Considerations for public health policies and intervention programs

Findings from our study suggest that there are gaps and differences in infant feeding recommendations coming from grandmothers and healthcare professionals. It is important to address these findings by considering educational and counseling interventions that promote breastfeeding without causing maternal guilt and that consider the existing traditional knowledges within a community [50]. Moreover, our study highlights the importance of including grandmothers in interventions given that they demonstrate an interest in learning from HCPs and sharing the information they learn with other mothers. Our study also demonstrates the lack of skillsets for HCPs to communicate accurate information regarding optimal breastfeeding practices, therefore, it would be beneficial to develop educational programs to address this lack of training.

There are two pedagogical models that could provide insightful strategies to address the gaps identified in this study. First, the pedagogical model from Colombia’s Universidad de Antioquia, which is based on a reflective-experiential process that deconstructs established truths and constructs new ways of approaching breastfeeding based on the needs of mothers and their families [22]. The “Trato Digno” model [51] is another one to consider because it invites participants to reflect on structural factors of racism, classism, and sexism within biomedicine in the context of Mexico’s culture. Elements from both models may help address structural factors that perpetuate the gaps between traditional and biomedical infant feeding recommendations.

### Limitations

This study should be interpreted taking into account the following limitations. First, the current study used secondary data of a previous study conducted by our team. As such, the primary objective of the original research was not to examine the differences in infant feeding recommendations between grandmother and healthcare professionals. Therefore, the interview questions were not specifically designed to address the question addressed in this article. Despite this limitation, as demonstrated in this article the secondary data analyses provided important insights into the different perspectives of grandmothers and HCPs. Secondly, there was limited information on how mothers negotiate receiving conflicting information from grandmothers and HCPs. Nevertheless, our study has now identified this knowledge gap as an important area for future research.

### Reflexivity statement

The research team conducting this study represents a diverse group of individuals with expertise in the areas of anthropology, medicine, nutrition, psychology, and public health; all of which have a shared interests in the topics of reproductive and infant health, particularly in infant feeding practices in the context of social inequities. All researchers are Mexican or of Mexican descent and have been privileged to access higher education and work within institutions in either Mexico or the United States. It is also important to acknowledge that none of them are from the communities who participated in this study. As such, the research team is aware that there are important differences and inequities between them and the participants, which are driven by colonialism and subsequent neocolonial social structures. For this reason, even though the study was designed to strongly represent the voices from the communities, the team acknowledges that its interpretation of results for the present study have been influenced by the research team’s social privileges, experiences, assumptions, and beliefs.

## Conclusions

The findings from our study highlight significant differences on recommendations between grandmothers and HCPs across infant feeding topics ranging from timing of introduction of other foods to breastfeeding duration. These different perspectives from major influential figures have the potential to generate confusion among mothers in deciding what to do or to whom they should listen. Another source of tension for mothers may be instigated by the legacies of colonialism within biomedicine, which continue to impact HCPs’ assumptions about communities they serve, the medical attention that mothers receive, and the messages delivered in the media. Given the long and ongoing colonial history of Mexico, it is imperative for future research on infant feeding practices to take into account this historical context. Moreover, given the influence of grandmothers on infant feeding practices, future public health programs that aim to improve breastfeeding rates should earnestly value and seek the inclusion of grandmothers.

## Supplementary Information

**Additional file 1.** Interview guides.

## Data Availability

The datasets during and/or analyzed during the current study available from the corresponding author on reasonable request.
