# Supplementary material for: Discrepancies in infant feeding recommendations between grandmothers and healthcare providers in rural Mexico
Source: Int Breastfeed J. 2022 Nov 22;17:77. doi: 10.1186/s13006-022-00518-0 (PMC9682729; doi:10.1186/s13006-022-00518-0)
Supplement: Supplementary file 1 — Additional file 1. Interview guides. [file 13006_2022_518_MOESM1_ESM.docx]

**Additional File 1**—Interview and Focus Group Guides Translated to English

**A. Demographic Questionnaire for Family Members**

1. What is your age (years)?
2. What was the last grade in school that she completed?
3. What is your occupation?
   1. Employed (specify occupation)
   2. Unemployed
4. Do you speak an indigenous language?
5. What type of home do you have (house, apt., etc.)?
6. Characteristics of the house:
   1. What kind of roof do you have?
   2. What kind of flat do you have?
   3. What kind of walls do you have?
   4. Do you have electricity?
   5. Do you have drinking water?
   6. Do you have a sewer system?
   7. Do you use gas / electricity / firewood?
7. Marital status:
   1. Single
   2. Widowed.
   3. Married
   4. Living with a partner
   5. Separated
   6. Divorced
8. Are you enrolled in *Oportunidades*/other social programs?
9. Who do you live with in your home?
10. How many children do you have?
11. How old are your children? (NOT FOR GRANDMOTHERS)

**B. Guide for Mothers**

1. How many children do you have? Is this your first child?
2. What is the age of your youngest child? What is their name?
3. Are you the primary caregiver for [NAME]? (who is in charge of feeding them, you’re your partner/family help, if another person – what do they think about how they feed the baby)
4. And what has motherhood been like for you?
5. Tell me about your pregnancy? (complications, consultations, breastfeeding consultations)
6. Tell me about your delivery? (Cesarean section/vaginal delivery, complications, colostrum, joint stay, first food and who fed the baby, how long did it take for colostrum, problems breastfeeding for the first time, problems with previous children, if mother has had problems: why did you continue breastfeeding)
7. How did you feed your baby from birth to 6 months? (plain water, tea, sickness, traditional practices)
8. How did you feed your baby after 6 months? (complications, insufficient milk)
9. In your experience, what practices have you seen other moms use to feed their babies? (Some moms give EBF for 6 months, what do you think? Breaking of traditional practices in young moms, fear of young moms, contradictions of information from doctors vs traditional practices)
10. Have you heard if there are benefits for mothers who breastfeed their babies? (who provided this info)
11. Do you work/plan to work or do activities outside the home that could keep you away from [NAME] for most of the day? And how do you plan to feed your baby while you are working?

**C. Guide for Grandmothers**

1. Are you a maternal/paternal grandmother?
2. How many grandchildren do you have?
3. What is the age of your youngest grandchild? What is their name?
4. Do you see/care for [NAME] frequently? (Where does the grandchild live?)
5. And how was that experience of caring for him/her?
6. And how was your daughter/daughter-in-law's pregnancy? (complications, consultations, information during breastfeeding consultations)
7. And how was the delivery of your daughter/daughter-in-law? (Cesarean section/vaginal delivery, complications, colostrum, joint stay, first food and who, how long did it take for colostrum)
8. Could you tell me how your grandchild has been fed/was fed in the first 6 months? (plain water, tea, sickness, traditional practices)
9. Could you tell me how your grandchild has been/was fed after 6 months?
10. From your experience, what is the best way to feed babies? And what recommendations have you given to your daughter/daughter-in-law?
11. What are the main problems or inconveniences that your daughter-in-law/daughter has gone through to feed your grandchild? What did they do to fix it? (insufficient milk)
12. In your experience, what practices have you seen other moms use to feed their babies? (Some moms give EBF for 6 months, what do you think? Breaking of traditional practices in young moms, fear of young moms, contradictions of information from doctors vs traditional practices)
13. Have you heard if there are benefits for mothers who breastfeed their babies? (who gave info)

**D. Guide for Healthcare Providers**

1. What protocols are followed here in the clinic/hospital during pregnancy? (consults, information about breastfeeding)
2. What protocols are followed here in the clinic/hospital after delivery? (Cesarean section/vaginal delivery, complications, colostrum, joint stay, first food and who feeds the baby, colostrum delays, problems breastfeeding for the first time)
3. What recommendations do you give mothers about feeding children under two years of age? (plain water, tea, illness, traditional practices, insufficient milk)
4. How do you explain the importance of breastfeeding to mothers? (benefits for mom/baby)
5. What influence does the infant feeding industry have on mothers' decisions to feed their baby?
6. Other than these recommendations, do you know of any cultural/religious practices or advice in the community about infant feeding?
7. What are the main barriers that you identify for mothers/fathers to adhere to your recommendations?
8. Demographic questions
   1. Age
   2. Sex
   3. Training: position/specialty
   4. What courses did you take on infant feeding?
   5. Any updated training
   6. Homogeneous training/recommendations between colleagues
   7. Time in this position
   8. Time working in this community
